# Aurora kinase B inhibition reduces the proliferation of metastatic melanoma cells and enhances the response to chemotherapy

**DOI:** 10.1186/s12967-015-0385-4

**Published:** 2015-01-27

**Authors:** Letizia Porcelli, Gabriella Guida, Anna E Quatrale, Tiziana Cocco, Letizia Sidella, Immacolata Maida, Rosa M Iacobazzi, Anna Ferretta, Diana A Stolfa, Sabino Strippoli, Stefania Guida, Stefania Tommasi, Michele Guida, Amalia Azzariti

**Affiliations:** Clinical and Preclinical Pharmacology Laboratory, National Cancer Research Centre Istituto Tumori Giovanni Paolo II, Viale O. Flacco,65, 70124 Bari, Italy; Department of Basic Medical Sciences, Neurosciences and Sense Organs, University of Bari, P.zza Giulio Cesare, 70124 Bari, Italy; Medical Oncology Department, National Cancer Research Centre Istituto Tumori Giovanni Paolo II, Viale O. Flacco,65, 70124 Bari, Italy; Unit of Dermatology and Venereology, University of Bari, P.zza Giulio Cesare, 70124 Bari, Italy; Molecular Genetics Laboratory, National Cancer Research Centre Istituto Tumori Giovanni Paolo II, Viale O. Flacco,65, 70124 Bari, Italy

**Keywords:** Melanoma, Barasertib, Vemurafenib, Nab-paclitaxel, BRAF status

## Abstract

**Background:**

The poor response to chemotherapy and the brief response to vemurafenib in metastatic melanoma patients, make the identification of new therapeutic approaches an urgent need. Interestingly the increased expression and activity of the Aurora kinase B during melanoma progression suggests it as a promising therapeutic target.

**Methods:**

The efficacy of the Aurora B kinase inhibitor barasertib-HQPA was evaluated in BRAF mutated cells, sensitive and made resistant to vemurafenib after chronic exposure to the drug, and in BRAF wild type cells. The drug effectiveness has been evaluated as cell growth inhibition, cell cycle progression and cell migration. In addition, cellular effectors of drug resistance and response were investigated.

**Results:**

The characterization of the effectors responsible for the resistance to vemurafenib evidenced the increased expression of MITF or the activation of Erk1/2 and p-38 kinases in the newly established cell lines with a phenotype resistant to vemurafenib. The sensitivity of cells to barasertib-HQPA was irrespective of BRAF mutational status. Barasertib-HQPA induced the mitotic catastrophe, ultimately causing apoptosis and necrosis of cells, inhibited cell migration and strongly affected the glycolytic metabolism of cells inducing the release of lactate. In association i) with vemurafenib the gain in effectiveness was found only in BRAF(V600K) cells while ii) with nab-paclitaxel, the combination was more effective than each drug alone in all cells.

**Conclusions:**

These findings suggest barasertib as a new therapeutic agent and as enhancer of chemotherapy in metastatic melanoma treatment.

**Electronic supplementary material:**

The online version of this article (doi:10.1186/s12967-015-0385-4) contains supplementary material, which is available to authorized users.

## Background

Metastatic melanoma (MM) is amongst the most resistant solid tumors to chemotherapy, radiotherapy, and prior investigational agents. Prior to 2011, only few chemotherapeutic agents in common use had achieved regulatory approval for treatment of MM and none resulted in significantly improved survival. Robust advances in our understanding of the molecular biology of melanoma and on the complex role of host immunity have opened the field of melanoma therapy to molecularly targeted agents and to immunotherapy unlocking the immune response, respectively. Emerging data from recently completed clinical trials and preliminary data from ongoing studies testing novel targeted agents suggest BRAF inhibitors vemurafenib and dabrafenib in patients carrying V600E mutation of BRAF gene and ipilimumab, a human monoclonal antibody that blocks the activity of CTLA-4 antigen inducing a modulation of T-cell activity as new therapeutic options [1]. Patients treated with a BRAF inhibitor had a clinically significant prolongation of survival over 13-16 months as a first line therapy [2,3] and rapid tumour regression; however, the majority of them acquires resistance to therapy and relapses very rapidly [4]. So far, several mechanisms of resistance involving different molecular pathways have been described after vemurafenib such as the activation of the proliferation and survival pathways, the amplification of MITF and/or CDK-2 and so on and numerous are the attempts that are being explored to overcome the resistance [5]. One of recent approach followed by most scientists is to block the MAPK pathway, which is activated in the establishment of resistance to BRAF inhibitors. This therapeutic approach involves the use of MEK inhibitors, but unfortunately the published results are not as promising as hoped by scientific audience [6]. Very promising results are being obtained with the combination therapy anti-BRAF plus anti-MEK [7]. Frequent is the question whether there is a role for chemotherapy in MM [8]. Recently, new chemotherapeutic molecules have been investigated and some of them demonstrated high activity in MM. Over all is Abraxane, a solvent-free albumin-stabilized nanoparticle formulation of paclitaxel which has been investigated in different cancers reporting very positive results [9]. The preliminary results of a large, open-label multicenter phase III trial, recently concluded and comparing abraxane vs. dacarbazine in previously-untreated patients with MM, have confirmed the positive results of previous phase II studies with clinically meaningful benefit in both BRAF mutated and wild type patients with acceptable toxicity, hence it should be considered among the treatment options for MM patients treatment [10-12]. Although in preclinical investigations, several Aurora kinases inhibitors, such as MLN8054, PHA-739358, VE-465, ZM447439, SNS-314 and JNJ-7706621, have been utilized in preclinical studies on melanoma models, demonstrating to inhibit cell proliferation, to induce apoptosis, and to inhibit cell migration in melanoma as respect to melanocytes [13-17], only one Aurora A kinase inhibitor (MLN8237) is in a Phase II clinical trials for patients with unrespectable Stage III-IV melanoma (clinicaltrials.gov). Recently, literature data reported the promising opportunity to combine the inhibition of Aurora A kinase with that of BRAF or MEK in BRAF mutated or wild type MM models [13], while no evidence currently exist testing the combination of Aurora kinases inhibitors with chemotherapy in melanoma treatment.

In this report, we explored the reliability of targeting Aurora B kinase which plays a crucial role in cell mitosis [18]. The Aurora B kinase trough the phosphorylation of histone H3 and by forming the chromosomal passenger complex (CPC) together with survivin, INCENP and borealin, allows the segregation of chromatids at mitosis and the corrected cytokinesis [19].

Therefore inhibiting Aurora B kinase results in the impairment of cellular mechanisms leading to mitosis and tumor proliferation. The use of Aurora B kinase inhibitors for therapeutic uses is also suggested from the observation that the expression and activity of this protein is increased during melanoma progression [20-22]. Several small molecules, inhibitors of Aurora B kinase have been developed and are currently in early clinical evaluation for treatment of various tumor pathologies. They include barasertib, the drug used in our study [23-25]. Barasertib (AZD1152, kindly provided by AstraZeneca) is a dihydrogen phosphate prodrug of a pyrazolo quinazoline Aurora kinase inhibitor [AZD1152] that is rapidly converted into the active moiety barasertib-HQPA following parenteral administration; then the active metabolite is a highly potent and selective inhibitor of Aurora B kinase [26]. Currently, barasertib is ongoing in a phase I/II trials for the treatment of patients with Diffuse Large B-cell Lymphoma and in a phase II/III trials alone and in combination with low dose of cytosine arabinoside in acute myeloid leukaemia patients (SPARK-AML1) (www.clinicaltrials.gov). Here, we evaluated the anti-tumor effects of barasertib-HQPA in MM cell models carrying BRAF(V600E) or BRAF(V600K) mutations and wild type BRAF in order to evaluate the efficacy of barasertib in cells responding to anti-BRAF vemurafenib and do not, respectively. We found that barasertib was very effective in inhibiting tumor proliferation of both BRAF mutated and wt cells. Interestingly, as a consequence of the exposure to barasertib we observed that the higher the efficacy of the drug the higher was the release of lactate, hence suggesting that it could be utilized as a biomarker of response to barasertib in MM cells. In addition we combined barasertib-HQPA with vemurafenib in order to gain further knowledge on the possibility to overcome resistance to vemurafenib by targeting Aurora B kinase. Moreover we combined barasertib-HQPA with nab-paclitaxel in order to assess the optimal combination schedule with chemotherapy and provide evidence on an innovative therapeutic approach to be used for the treatment of melanoma.

## Methods

### Drugs and chemicals

Barasertib-HQPA was provided by AstraZeneca Pharmaceuticals (Macclesfield, U.K.). Stock solutions of Barasertib-HQPA were prepared at 20 mM in DMSO and stored in aliquots at –20°C. Further dilutions were made in medium supplemented with 10% foetal bovine serum, 2 mM glutamine, 50,000 UL^−1^ penicillin and 80 μM streptomycin.

### Cell lines

The Hmel-1, MBA72 cell lines was obtained from bioptic samples of MM and genotyping for NRAS and BRAF [27,28]. Hmel-1 showed BRAF mutation in V600K and MBA72 in V600E, both in heterozygosis. HBL, LND1 cell lines, both wild type (wt) for BRAF, are a gift of Prof. G. Ghanem, University of Bruxelles. All cell lines were wt for NRAS. Cells were cultured in vitro in D-MEM supplemented with 10% foetal bovine serum, 100 U/ml penicillin, 100 μg/ml streptomycin in a humidified incubator at 37°C with an atmosphere containing 5% CO_2_.

### Cell imaging

Cells were exposed to 30 and 300 nM barasertib-HQPA for 1-3 days and their shape were analysed by light inverted microscopy.

### Cell proliferation assay

Determination of cell growth inhibition was performed using the 3-[4,5-dimethylthiazol-2-yl]-2,5-diphenyltetrazoliumbromide (MTT) assay and by cell counting. The MTT assay for the determination of the concentration responsible for 50% inhibition of cell growth (IC_50_) was performed as described in Porcelli et al. [29]. The IC_50_ was defined as the drug concentration yielding a fraction of affected (no surviving) cells = 0.5, compared with untreated controls and was calculated utilising CalcuSyn ver.1.1.4 software (Biosoft, UK).

For cell count determination, cells were exposed to barasertib-HQPA alone or in combination with vemurafenib or nab-paclitaxel, harvested in trypsin and cells were counted. Barasertib -HQPA was given at 30 and 300 nM, vemurafenib at the IC_50_ concentration and nab-paclitaxel at 50 nM which induced after 3 days about the 50% of cells death. The Combination Index (CI) was calculated by CompuSyn for Drug Combinations and for General Dose-Effect Analysis (ComboSyn, Inc. 599 Mill Run, Paramus, NJ, 07653, USA). CI < 1 means synergism; CI > 1 means antagonism; CI = 1 means additivity.

### Cell cycle analysis

After two wash steps in ice-cold PBS (pH 7.4), cells were fixed in 4.5 ml of 70% ethanol and stored at -20°C. For the analysis, the pellet was resuspended in PBS containing 1 mg/ml RNase, 0.01% NP40 and 50 μg/ml propidium iodide (PI) (Sigma). After an incubation time of 1 hour in ice, cell cycle determinations were performed using a FACScan flow cytometer (Becton Dickinson), and data were interpreted using the CellQuest software, provided by the manufacturer.

### Cell apoptosis assay

Apoptosis detection was further investigated by the Cell Death ELISA^PLUS^ kit (Roche Molecular Biochemicals, Milan, Italy). The test is based on the detection of mono- and oligonucleosomes in the cytoplasmic fraction of cell lysates by biotinylated anti histone-coupled antibodies, and their enrichment in the cytoplasm is calculated as the absorbance of sample cells/absorbance of control cells. The enrichment factor was used as a parameter of apoptosis and shown on the Y-axis as mean ± SE. Experiments were performed according to manufacturer’s instructions.

### Lactate dehydrogenase activity

Cells undergoing necrosis typically exhibit rapid swelling, loss of membrane integrity, and release of lactate dehydrogenase (LDH). This enzyme activity into the culture medium was measured as described by Bernt and Bergmeyer [30] and expressed as percent of maximum LDH released 3 days after drug(s) exposure [31]. An aliquot (100 μl) of culture medium (5×10^4^ cells/1 ml culture medium) was added to 2 ml of 50 mM Tris–HCl buffer pH 7.4 in the presence of 0.2 mM NADH. The assay reaction was started by adding 0.6 mM pyruvate.

### Wound healing assay

Confluent monolayer of MM cells were wounded and treated with barasertib-HQPA (30-300 nM) or left untreated (control). The plates were photographed 0, 24 and 48 hours post-wounding. Cell migration was quantified by counting the wound width after the plates were treated, utilizing ImageJ® analysis software. Results are given as migration length and are accounted for by the average per field ± S.D. of three independent experiments.

### Western blot analysis

Protein extracts were obtained by homogenization in RIPA buffer (0.5 M NaCl, 1% Triton X100, 0.5% NP40, 1% deoxycolic acid, 3.5 mM SDS, 8.3 mM Tris HCl pH 7.4, 1.6 mM Tris base) and treated with 1 mM phenylmethylsulfonyl fluoride (PMSF). Total proteins were measured and analyzed as described in [32]. 50 μg were electrophoretically separated on 10% acrylamide gel (SDS–PAGE by Laemli). Signal was detected by chemoluminescence assay (ECL-Plus, Amersham Life Science, UK). Expression levels were evaluated by densitometric analysis using Quantity One software (Biorad, Hercules, CA) and β-actin expression levels were used to normalize the sample values.

### Antibodies

All monoclonal antibodies utilised were provided by Cell Signalling-USA and Sigma-Aldrich, St. Louis, MO-USA. A mouse-HRP and a rabbit-HRP (Amersham Pharmacia Biotech, Upsala Sweden) were used as secondary antibody.

### Fluorescence immunocytochemistry

Cells were seeded onto coverslips. After overnight incubation, they were fixed in 3.7% paraformaldehyde, washed and permeabilized with 0.1% Triton X-100. Nuclei were counterstained with 0.5 mg/ml 4′,6-diamidino-2-phenylindole (DAPI). The images were captured using a fluorescence microscope (Olympus BX40), equipped with X20 objective with a SenSys 1401E-Photometrics charge-coupled device camera. FITC was excited using the 488 laserline and DAPI using the 568 laserline.

### Extracellular lactate level

The amount of lactate in the cell medium was estimated following spectrophotometrically NADH oxidation at 340 nm as described in Pacelli et al. [33].

### Statistical analysis

All in vitro experiments were performed in triplicate, and results have been expressed as the mean ± standard deviation (SD), unless otherwise indicated. Statistical differences of in vitro and in vivo data were assessed by ANOVA, followed by the Student-Newman–Keuls test. P-values lower than 0.05 were considered significant. Statistical analyses were performed using the GraphPad Prism software package version 5.0 (GraphPad Software Inc., San Diego, CA, USA).

## Results

### Characterization of vemurafenib sensitivity in function of BRAF status

Sensitivity to vemurafenib was assessed in a panel of 4 cell lines: LND1 and HBL cells which have BRAF wild type (w.t.) and MBA72 and Hmel-1 cells carrying mutations V600E and V600K in BRAF, both known to be responsible for high responsiveness to vemurafenib in patients at least at the beginning of the therapy [27,28,1,34]. As expected, cells with mutated-BRAF were sensitive to vemurafenib showing an IC_50_ ranging between 3 and 5 μM; the IC_50_ value increased of 10-times in the BRAF w.t. cells (see Additional file 1: Figure S1(A) and Table 1). In order to mimic the induction of resistance to vemurafenib observed in MM patients treated with this drug, all the cells carrying BRAF mutations were chronically (21 days) treated with vemurafenib. After 21 days of exposure, the IC_50_ of still proliferating cells increased of about 10 and 5 times in MBA72 and Hmel-1, respectively (Table 1). These newly established cell lines were named MBA72R and Hmel-1R.

**Table 1:** **Vemurafenib and barasertib activity evaluation in living cell lines**

**Cell line**	**BRAF status**	**3 days-vemurafenib**	**3 days-barasertib-HQPA**
		**IC**_**50**_ **(μM)**	**IC**_**50**_ **(nM)**
MBA72	V600E	3.2 ± 0.6	305 ± 4
Hmel-1	V600K	5.5 ± 0.4	311 ± 6
LND1	wild type	32.2 ± 1.1	307 ± 7
HBL	wild type	37.3 ± 0.9	315 ± 11
MBA72R	V600E	33.5 ± 0.5	367 ± 10
Hmel-1R	V600K	25.8 ± 0.5	184 ± 8

### Determinants of induced-resistance to vemurafenib in MBA72R and Hmel-1R

The onset of resistance to vemurafenib has been extensively studied and various cellular determinants responsible for this have been identified [5,35].

A preliminary screening of the biomarkers responsible for drug resistance in the two cell lines, and MBA72R and Hmel-1R, was conducted focusing on the involvement of i) the determinants of cell cycle progression by analysis of its perturbation, ii) Akt, Erk1/2 and p-38, activated as a consequence of the induction of resistance to vemurafenib, and finally it was also considered the possible involvement of MITF.

**Figure 1:**
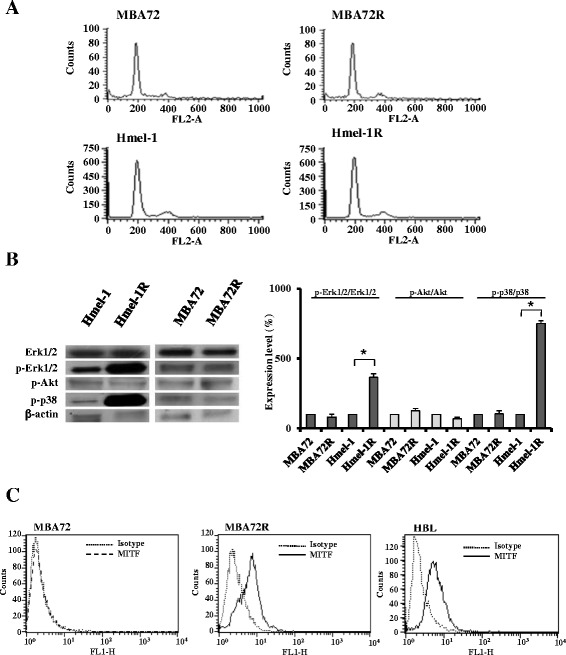
**Determinants of resistance to vemurafenib. A**. Cell cycle progression of the two pair of cells, MBA72/ MBA72R and Hmel-1/Hmel-1R, were determined as described in M&M section. **B**. p-Erk1/2, Erk1/2, p-Akt, Akt, p-p38 MAPK and p38 MAPK were analysed in cells by western blotting and protein amount determined by densitometry analysis. Histograms are means of at least three different experiments. *p < 0.05 vs untreated cells. **C**. The expression level of MITF was determined by FCM in MBA72, MBA72R and HBL as respect to the isotype.

In Figure 1A, histograms of cell cycle progression in the pairs of cell lines (MBA72 vs MBA72R and Hmel-1 vs Hmel-1R) are reported in which no evident changes in cell cycle progression are present suggesting the non-involvement of cell cycle determinants as responsible for vemurafenib-resistance. In Figure 1B, the western blotting characterization of pAkt/Akt, p-p38/p38 MAPK and pErk1/2/Erk1/2 are reported; Akt did not show any statistical variation from baseline level while Erk1/2 and p38 MAPK were activated only in Hmel-1R with an increase of pErk1/2 and p-p38 MAPK of 350 and 750% as respect the naïve cells. In Figure 1C, MITF determination by flow cytometry is showed with an increase of this transcription factor only in MBA72R (3.58 folds higher than in MITF negative-naïve cells) and the expression level is similar to that in HBL cells (about 2.19 folds higher that the negative cells), intrinsically resistant to vemurafenib. Thus, our results demonstrated that the chronic exposure to vemurafenib induced a decrease of drug sensitivity in function of the activation of Erk1/2 and p38 MAPK and the increase of MITF expression level in Hmel-1R and MBA72R, respectively.

### Barasertib-HQPA modifies cellular morphology and inhibits cell growth in function of drug concentration

Barasertib-HQPA increased cell size and consequently, only a direct counting of cells after drug(s) treatment provides a correct analysis of its cytotoxicity [26].

All cell lines were incubated in a range from 3 nM to 3 μM barasertib-HQPA for 3 days and the cell count showed that this inhibitor reduced cell proliferation in function of concentration (Additional file 1: Figure S1 and Table 1). The IC_50_ values are about 300 nM in all cells with the exception of Hmel-1R which resulted a little more sensitive as respect to the naïve cells. Prolonged drug exposure (6 days) was more effective in BRAF wt cells compared to BRAF-mutated ones and the exposure to barasertib-HQPA for 3 days followed by 3 days-drug wash out induced a marked recover of cell growth only in MBA72 (Additional file 1: Figure S1).

All following experiments were carried out with barasertib-HQPA at 30 and 300 nM, the highest concentration is comparable to the IC_50_s and the lowest could provide evidences on the efficacy of the drug at subtoxic concentration.

**Figure 2:**
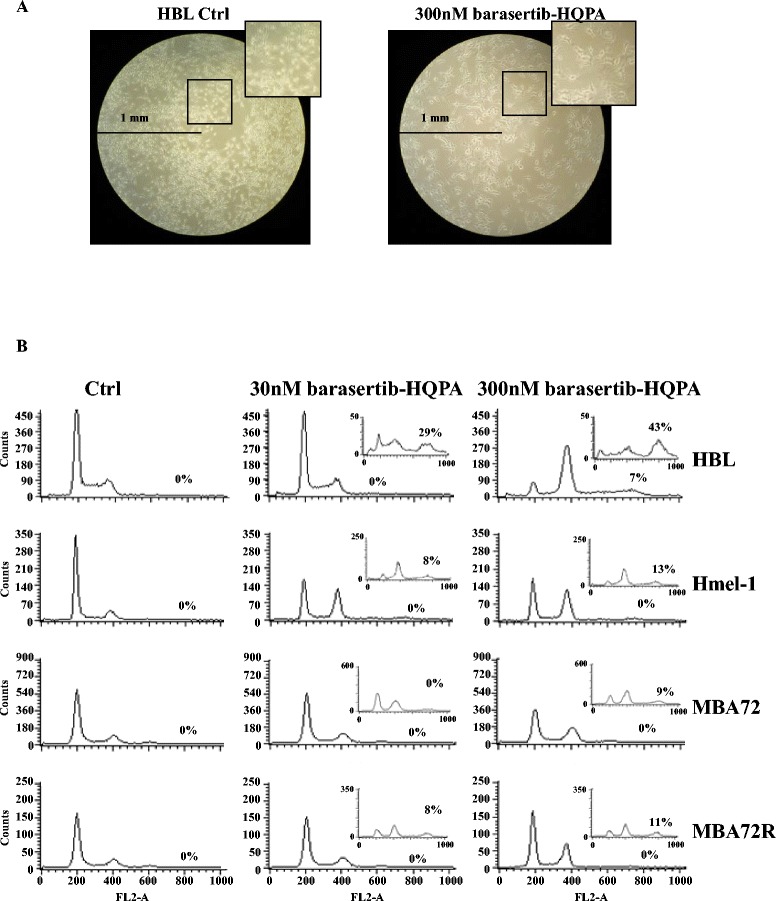
**Morphological changes and cell cycle perturbation by barasertib-HQPA exposure. A**. Increase in HBL cell size induced by barasertib-HQPA exposure at 300 nM by light inverted microscopy. **B**. Cells were incubated, for one and three days, with barasertib-HQPA (30 and 300 nM) and the cell cycle was analysed by flow cytometry analysis as described in M & M section. Experiments were performed in triplicate obtaining similar results and histograms are representative for all of them. Results of 3 days-exposure are reported in the inserts.

In Figure 2A cellular morphology after treatment with the Aurora B kinase inhibitor in HBL1 cells is reported and is representative of the marked phenotypical alterations obtained treating melanoma cells with this drug; the incubation with 300 nM barasertib-HQPA (for 3 days) caused the formation of giant cells with irregular shapes, suggesting a failure of cytokinesis [36].

### Barasertib-HQPA perturbs cell cycle progression

The marked reduction of cell growth may be justified by a strong perturbation of cell cycle progression. This hypothesis has been investigated in all melanoma cell lines in function of drug concentration and exposure time. In Figure 2B, only results obtained in HBL, Hmel-1 and in the pair MBA72/MBA72R are reported and the cell cycle progression of HBL and MBA72R are similar to those obtained for LND1 and Hmel-1R, respectively.

In BRAF wt cells, 1 day exposure to 30-300 nM barasertib-HQPA induced cell cycle arrest in mitosis with a strong accumulation of 4N cell population. After 3 days of treatment cells undergone an additional round of DNA duplication while failing cytokinesis, then 8N cells population started accumulating in a range from 8% in BRAF mutated to 43% in BRAF wt cells (inserts in Figure 2B). The behavior was similar, though less marked in BRAF-mutated cells, both in cell lines sensitive and resistant to vemurafenib. Then, barasertib-HQPA induced the formation of polyploid cells depending on drug concentration and time exposure.

### Barasertib-HQPA induces mitotic catastrophe

**Figure 3:**
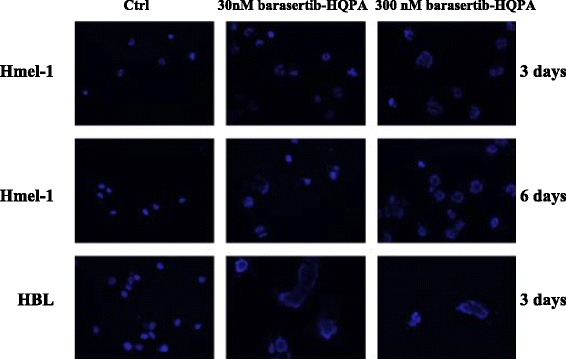
**Modification of nuclear morphology following exposure to barasertib-HQPA.** BRAF-mutated melanoma cells were exposed to 30 and 300 nM barasertib-HQPA and after three and six days, polynucleate cells were evidenced by ICC (blue: DAPI).

Barasertib-HQPA induced the accumulation of 4N and 8N cell population thus, the hypothesis that this drug may be an inducer of mitotic catastrophe was investigated. Mitotic catastrophe is a mechanism of cell death characterized by the occurrence of aberrant mitosis with the formation of large cells that contain multiple nuclei, which are morphologically distinguishable from apoptotic cells. Nuclear morphology after drug treatment was determined by DAPI staining and, as shown in Figure 3, cells exhibited pronounced changes in nuclear morphology and chromatin organization with visible multinucleated cells. The phenomenon increased in function of drug concentration but not of exposure timing and is irrespective of BRAF mutational status. This evidence, together with the appearance of giant cells with an unusual shape typical of cells which failed cytokinesis, suggests that the mitotic catastrophe may be a mediator of cell death activated by barasertib-HQPA in such MM models.

### Barasertib-HQPA induced apoptosis and necrosis

In a previous study, we demonstrated that the inhibition of Aurora B kinase activity by treatment with barasertib-HQPA induces apoptosis [26]. Here, the ability of this drug to induce cell death through various mechanisms was investigated. In addition to mitotic catastrophe, suggested in the previous paragraph, the induction of apoptosis and necrosis was evaluated by the DNA laddering and the LDH activity assay, respectively [37].

**Figure 4:**
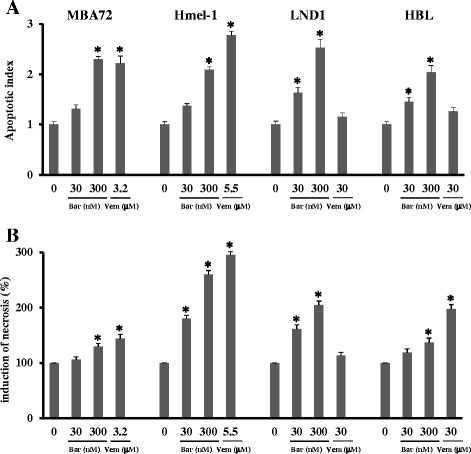
**Effect of barasertib-HQPA and vemurafenib on apoptosis and necrosis.** Melanoma cells were treated with 30 and 300 nM barasertib-HQPA (Bar) and vemurafenib (Vem) at IC_50_s. **(A)** Apoptosis was determined by Cell Death ELISA assay and **(B)** Necrosis by LDH activity determination and expressed as percentage respect to the untreated cells. *p < 0.05 vs untreated cells.

In Figure 4A, apoptosis induction by barasertib-HQPA or vemurafenib is reported and the first is responsible for programmed cell death increasing in function of drug concentration; conversely, vemurafenib induced apoptosis only in BRAF mutated cells as previously showed. In BRAF wt cells, vemurafenib did not activate this kind of death and the mechanism responsible for this lack of activity will be further investigated. Necrosis was determined by the analysis of LDH activity [30] and our data suggest that, in the presence of 30 and 300 nM barasertib-HQPA as well as of vemurafenib, cell death occurred via apoptosis and via necrosis except in HBL (Figure 4B). This increased LDH activity was depended on drug concentration. To explain the different behavior of HBL compared to LND-1 cells, the hypothesis that will be verified in the next future is that a low intracellular concentration of ATP might be responsible for the switch from apoptosis to necrosis, as suggested by Lemasters [38].

### Barasertib-HQPA activity was independent from survival and proliferation pathway

To explore the cellular pathways involved in determining barasertib-HQPA activity, the expression level of Erk1/2, Akt and p-38 in their phosphorylated forms as well as total ones were investigated by Western Blotting (Additional file 2: Figure S2). Results evidenced the absence of drug ability to modulate the basal forms of all proteins (not shown). In agreement with the involvement of Erk1/2 with the establishment of drug resistance [5], this protein was found activated after only 3 day-vemurafenib exposure in BRAF wt cells, which are resistant to vemurafenib perhaps for the ability of this drug to rapidly induce the phosphorylation of Erk1/2, but not in mutated cells, intrinsically sensitive to the BRAF inhibitor.

### Barasertib-HQPA inhibits cell migration

**Figure 5:**
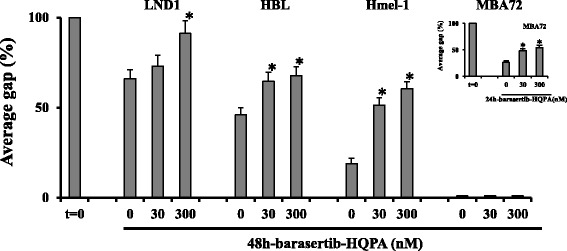
**Barasertib-HQPA ability to modulate cellular motility.** Wound healing assay was performed at 24-48 h with 30 and 300 nM barasertib-HQPA. Cell migration was quantified with ImageJ® analysis software and results are expressed as percentage of average gab vs drug concentration. *p < 0.05 vs untreated cells.

The anti-invasive effects of barasertib-HQPA were investigated on cellular motility by wound healing assay. A quite complete wound healing was evident when untreated cells were incubated at 37°C for 48 h conversely, monolayers treated with barasertib-HQPA, at 30 and 300 nM, showed clear wound width after 48 h in all cellular panels. Images and the quantified average gaps obtained in BRAF wt and mutated cells are reported in Additional file 3: Figure S3 and Figure 5, respectively. These results confirmed the ability of this compound to inhibit cell migration in a concentration-dependent way in this MM model.

### Barasertib-HQPA increases extracellular lactate levels

**Figure 6:**
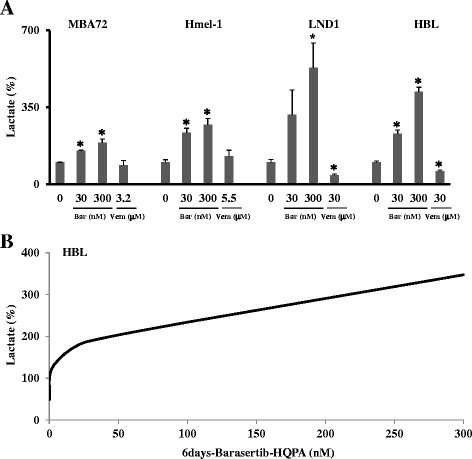
**Effect of barasertib-HQPA and vemurafenib on lactate production.** In **A**, extracellular lactate level was measured in the growth medium of cells exposed to either vehicle or barasertib (Bar) or vemurafenib (Vem) after 3 days. Data are means ± SEM and significance was calculated with Student’s t test; *p < 0.05 vs untreated cells. In **B**, the curve of the release of lactate are shown in function of barasertib-HQPA concentration (3, 30, 300 nM) and after 6 days of drug exposure.

The lactate was assayed as described in Methods and in all cells; the 3 days-exposure to barasertib-HQPA induced a significant concentration-dependent increase of the extracellular lactate levels mainly in BRAF wt cells conversely, vemurafenib reduced it only in the same model. (Figure 6A). In HBL cells (BRAF wt), the release of lactate in the medium after exposure to barasertib-HQPA was made using a wider range of concentrations and increasing the time of exposure to 6 days. The results showed a progressive increased release of lactate in function of the concentration and exposure time in HBL cells (Figure 6B).

### Barasertib-HQPA plus vemurafenib, a promising opportunity?

The first combined approach explored in this study was the administration of barasertib-HQPA plus vemurafenib with the aim to restore the sensitivity to vemurafenib.

Unfortunately, after the simultaneous exposure to barasertib-HQPA plus vemurafenib in MBA72R and Hmel-1R the inhibition of cell proliferation remained the same induced by barasertib alone (Additional file 4: Figure S4 A).

The effectiveness of three schedules, i.e. barasertib-HQPA together, before and after, was investigated in the responsive cell lines MBA72 and Hmel-1 which showed a different response. In MBA72 cells, harboring the mutation BRAF(V600E), the combination of the two drugs in all schedules was not effective, suggesting an antagonism between the two drugs. In Hmel-1, harboring the mutation BRAF(V600K), the sequential schedules were more effective than each drug alone conversely, the effectiveness of the combination did not increase when the two drugs were given simultaneously (Additional file 4: Figure S4 B). Thus, these data suggest to further explore whether the mutation BRAF V600K may be responsible for the increased effectiveness of the two drugs in combination.

### Barasertib-HQPA plus nab-paclitaxel: how relevant is the schedule?

The promising approach to combine new biological agent with conventional chemotherapeutics and the low number of chemotherapeutics utilised in this pathology suggested the combined administration of barasertib-HQPA plus nab-paclitaxel, a taxane derivative. The scientific rationale relays on the selective effect of each drug on mitotic machinery; paclitaxel arrests cells division by stabilizing microtubule polymers thereby disrupting the cellular machinery required for mitotic spindle assembly and barasertib inhibits Aurora B kinase, which forms the chromosomal passenger complex and allows ultimately a correct cytokinesis.

**Figure 7:**
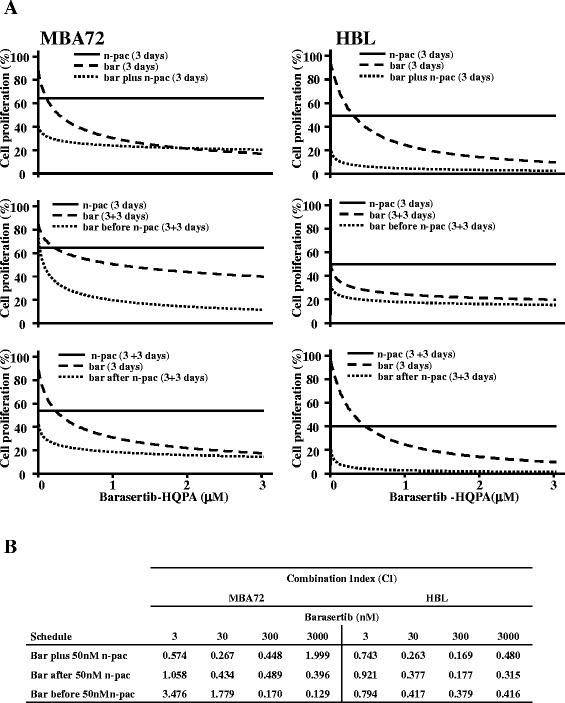
**Barasertib-HQPA plus nab-paclitaxel in melanoma cell panel.** MBA72 and HBL cells were incubated with 3, 30, 300 nM and 3 μM barasertib-HQPA (bar) plus 50nM nab-paclitaxel (n-pac) given in three schedules, simultaneous (3 days) and 3 days-barasertib-HQPA before or after 3 days-nab-paclitaxel. **A**. The survival of cells is reported as dose/effect plots. **B**. The quantification of the CIs of each drugs combination by CompuSyn software is summarized in the table.

The analysis of barasertib-HQPA and nab-paclitaxel in combination was performed utilising the first drug at four different concentrations (3, 30, 300 nM and 3 μM) and nab-paclitaxel at the fix dose of 50 nM. Three administration schedules were tested: simultaneous, barasertib-HQPA before and barasertib-HQPA after. Promising are all schedules utilized, in fact, either in BRAF wt cells and in mutated ones, the drugs combination induced a reduction of cell proliferation showing a synergic interaction (CI < 1) in quite all samples (Figure 7) suggesting the existence of a close interconnection between the pathways of action of the drugs. Indeed, the analysis of the nuclear morphology carried out on HBL cells, evidenced nuclear dysfunction which is consequent to mitotic abnormalities caused by each drug. Barasertib-HQPA induced the formation of big nuclei as a consequence of polyploidy and nuclear budding which is a biomarker of chromosomal instability; nab-paclitaxel resulted in the formation of mitotic aggregates of condensed chromosomes, conceivably due to the blockage of microtubule-dependent processes required for metaphase/anaphase transition. The combination of the two drugs resulted in a lethal effect, as all the nuclei in the observed specimens display necrotic and apoptotic like features (Additional file 5: Figure S5).

## Discussion

Aurora B kinase is increasingly overexpressed during melanoma progression with a concomitant enhancement of its activity, hence suggesting that it is a promising cellular target for personalized anticancer therapy in this disease [20-22]. Recently, Bonnet and coauthors provided evidences on the possibility to utilize barasertib, an Aurora B kinase inhibitor, as a new therapeutic strategy in both wild-type BRAF and vemurafenib-resistant BRAFV600E melanoma, stressing the idea that Aurora B kinase is a potential target in the therapy of MM [18].

Here, we provide preclinical evidence on the utilization of barasertib-HQPA in MM cell lines, including both BRAF wt and mutated cells and also two vemurafenib induced resistant cell lines. In addition we demonstrated that the inhibition of Aurora B kinase could improve the response to vemurafenib and to nab-paclitaxel, the latter recently included in clinical trials for treatment of MM.

All characterization was performed by taking into account the molecular characteristics of cells and results obtained suggested the utilization of barasertib in mono and multi-therapy in BRAF wt and mutated population.

The in vitro panel was characterized for vemurafenib sensitivity, confirming the effectiveness of this drug in the BRAF mutated cells conversely, it acted at ten-folds higher doses in the BRAF wt and in those which became resistant after chronic exposure to subtoxic doses of vemurafenib. A preliminary characterization of the cellular determinants responsible for the acquired-resistance to vemurafenib evidenced that the reactivation of Mek/Erk1/2 and p38 MAPK pathways might be involved in the establishment of resistance to the drug in Hmel-1, while the amplification of MITF was found in MBA72 cells after continuous exposure to vemurafenib. All these alterations have been already demonstrated to be directly related with the onset of the resistance to BRAF inhibitors [5,35,39,40].

All MM cells incubated with the Aurora B kinase inhibitor showed marked modification of the cellular morphology and a strong reduction of cells proliferation. Extending drug exposure to 6 days evidenced a difference between cells; BRAF wt cells were more sensitive than BRAF-mutated ones, both sensitive to vemurafenib and their resistant counterparts.

The importance of the identification of a predictive factor for a drug effectiveness is ubiquitously accepted, the scanty and contradictory evidences on biomarkers predicting the efficacy of Aurora kinase inhibitors reported in literature focused initially on CHFR, a mitotic checkpoint protein and p53, the inactivation of which leads to increased sensitivity to this class of drugs [41-43]. Recently, new technologies which highlighted genomic aberrations (point mutations, amplifications and rearrangements) have added new hypothesis. Simon and Roychowdhury suggested the amplification of the Aurora A Kinase (AURKA) in prostate and breast cancer [44]. According to the evidence that cells with high endoreduplication showed increased glycolytic metabolism with consequent increase of lactate release [45], in our models, barasertib-HQPA induced a marked increase of this metabolite in the medium in function of time and drug concentration. As the BRAF wt cells which released the highest level of lactate were the most sensitive to the drug, this suggested that the lactate release might predict drug effectiveness. The evidence that in BRAF mutated cells vemurafenib did not affect lactate levels while it was significantly reduced in BRAF wt cells is in disagreement with data reported in the literature in which a strict correlation between vemurafenib-induced p-Erk1/2 inhibition and reduction of glycolisis is hypothesized [46]. This discrepancy may lie in the different ability of the drug to affect pErk1/2 level with a strong increase or an absence of effect, in BRAF wt and mutated MM cells, respectively. Further investigations are warranted to highlight the relation between vemurafenib and glycolysis in such models.

The effectiveness of barasertib-HQPA in inhibiting cell proliferation was irrespective of BRAF mutation status. This suggested to investigate combination schedules of barasertib-HQPA with vemurafenib in order to restore and/or increase the sensitivity to vemurafenib in no longer sensitive cells. Our results showed that the Aurora B kinase inhibitor did not restore the sensitivity to vemurafenib in both resistant cells lines and that the two drugs were antagonist in cells harboring the BRAF(V600E) mutation. This result could be explained by the decrease of Aurora B kinase after vemurafenib exposure reported by other authors who suggested for such kinase a role as predictor factor for the response to BRAF inhibitors [18,20].

Thus, our data suggest that barasertib could be a promising approach for treatment of MM patients with BRAF wt, for whom no effective therapy are actually available, and for BRAF mutated MM patients as an alternative to vemurafenib. Moreover, the combination of barasertib-HQPA with vemurafenib resulted in a slight gain of effectiveness, irrespective of the schedule utilized, when the cell model harbors the mutation BRAF(V600K). This evidence, if supported by further investigation already in progress in our lab, could allow a better selection of MM population to treat with this innovative multidrug approach.

The investigation of the mechanism of action of barasertib-HQPA in our cell panel evidenced a marked G2/M phase cell accumulation associated with endoreduplication, increased DNA content, consequent death of cells, which occurred through mitotic catastrophe, apoptosis and necrosis induction. Moreover, barasertib-HQPA inhibited cell migration, showing to affect the invasive behavior of metastatic melanoma. Our results are in agreement with the ability of this compound to induce cell death by the activation of different mechanisms such as necrosis, apoptosis, mitotic catastrophe, senescence [26,47]. Further analysis will be focused on the possibility that senescence may be involved in determining the effectiveness of barasertib and Raf kinase Inihibitory Protein (RKIP) may be responsible for the crosstalk between Aurora B kinase pathway and Raf/MEK/Erk signaling in cells, in which the last is affected by the establishment of resistance to vemurafenib, and on the study of the crosstalk between the different pathways responsible for each type of cell death, with the aim of identifying how to switch from one to the other and optimize the clinical use of this drug [18,48]. The other explored therapeutic strategy was the combination of barasertib-HQPA plus nab-paclitaxel.

All the combinations with barasertib-HQPA and nab-paclitaxel resulted much more effective than each drug alone in inhibiting cell proliferation. This demonstrated that the combination schedule utilized is not relevant for treatment efficacy, most likely because each drug effect was anyhow synergic and resulted in an override of the mitotic arrest, with a more severely affected alignment of chromosomes and more aberrant nuclei with concomitant cell death induction.

## Conclusion

The evidence reported here further establishes Aurora B kinase as a therapeutic target in the treatment of both BRAF wild type and V600 mutated melanoma either vemurafenib responsive and no longer responsive melanoma. Targeting Aurora B kinase potentially has a dual antitumor role, by directly inhibiting migration and growth of cells and as enhancer of vemurafenib efficacy in BRAF(V600K) melanoma and of nab-paclitaxel based chemotherapy in BRAF wild type and mutated melanoma. In addition, through the combination study of barasertib with nab-paclitaxel we provide a proof of concept of the reliability of such combination suggesting administration schedules that properly validated in vivo and in rationally designed clinical trials might be a new efficient approach to treat melanoma.

## Additional files

Additional file 1: Figure S1. Vemurafenib and barasertib-HQPA activity in melanoma cell panel. A. Melanoma cells were incubated with vemurafenib, ranging between 0.1 and 100 μM and the survival of cells was determined using MTT assay. The results are showed as dose/cell growth inhibition plots of the mean of three different experiments, evidencing that mutated cells are more sensitive to the drug. B. Melanoma cells were incubated with Barasertib-HQPA, ranging between 3 nM and 3 μM and for increasing time. The survival of cells was determined using direct cell count. The results are showed as drug cell proliferation inhibition/dose plots of the mean of three different experiments, evidencing that BRAF wt cells are more sensitive to the drug than the BRAF mutated ones. Additional file 2: Figure S2. Effect of barasertib-HQPA and vemurafenib on cell transduction pathways. Both BRAF-mutated and wt melanoma cells were incubated with barasertib-HQPA (Bar) and vemurafenib (Vem) for 3 days. The protein extracts from all samples were analysed by western blot utilising the β-actin to normalize the values and are quantified as phosphorylated form as respect the total one. A. Bands of p-Erk1/2 expression level from all samples are reported together with the quantification. Histograms are means of at least three different experiments. *p < 0.05 vs untreated cells. *p < 0.05 vs untreated cells. Additional file 3: Figure S3. Barasertib-HQPA ability to modulate cellular motility. Wound healing assay was performed at 24-48 h with 30 and 300 nM barasertib-HQPA. Additional file 4: Figure S4. Barasertib-HQPA plus vemurafenib in mutated melanoma cells. A. MBA72R and Hmel-1R were incubated with 300 nM barasertib-HQPA (Bar) and/or Vemurafenib (Vem: at 3.2 and 5.5 μM in MBA72R and Hmel-1R, respectively) for 3 days. The proliferating cells were determined using the direct cell count. Histograms are means of at least three different experiments. B. MBA72 and Hmel-1 cells were incubated with barasertib-HQPA plus vemurafenib given in three schedules, simultaneous (3 days) and 3 days-barasertib-HQPA (3, 30, 300, 3000 nM) before or after 3daysvemurafenib, and the survival of cells was determined using the direct cell count. Additional file 5: Figure S5. Barasertib-HQPA plus nab-paclitaxel in melanoma cell panel. HBL cells exposed to 300 nM barasertib-HQPA and 50 nM nab-paclitaxel, alone or in combination, and polynucleate cells were evidenced by ICC (blue: DAPI).
