# Supplementary figures and images for: Aurora kinase B inhibition reduces the proliferation of metastatic melanoma cells and enhances the response to chemotherapy
Source: J Transl Med. 2015 Jan 27;13:26. doi: 10.1186/s12967-015-0385-4 (PMC4314759; doi:10.1186/s12967-015-0385-4)

Fig. 1S

A

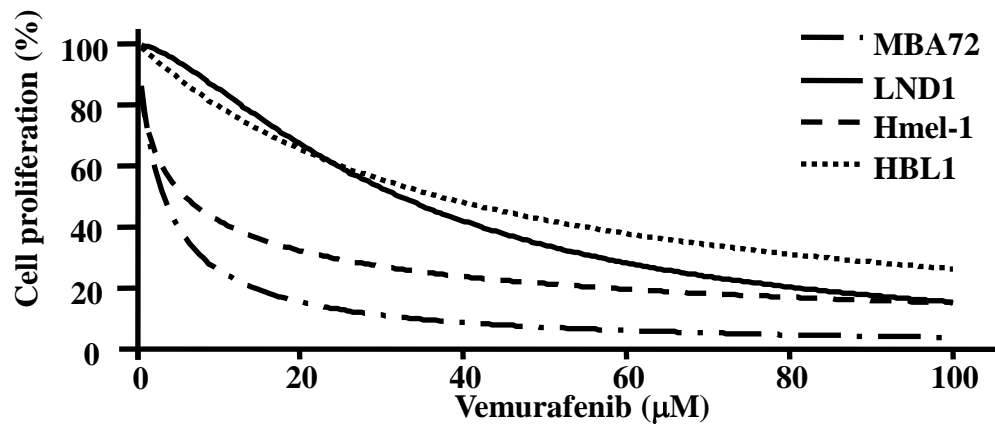

B

MBA72

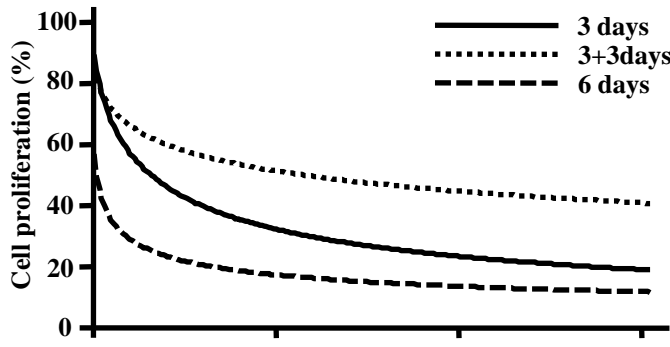

MBA72R

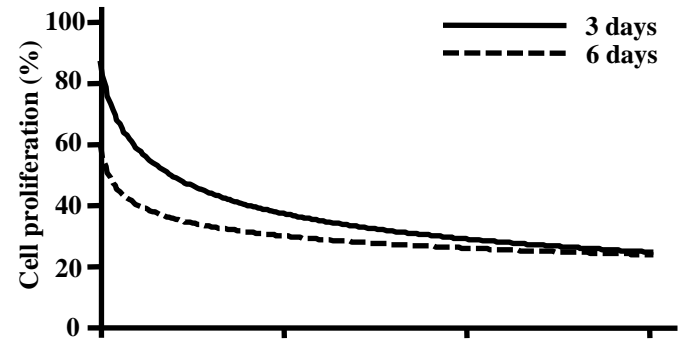

Hmel-1

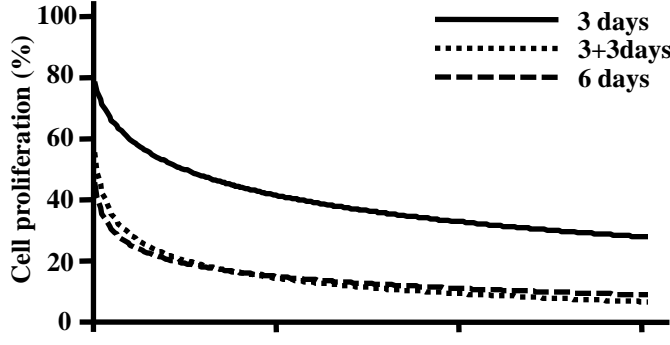

Hmel-1R

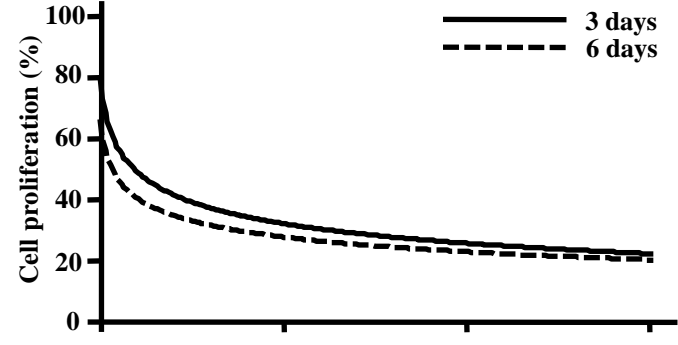

HBL

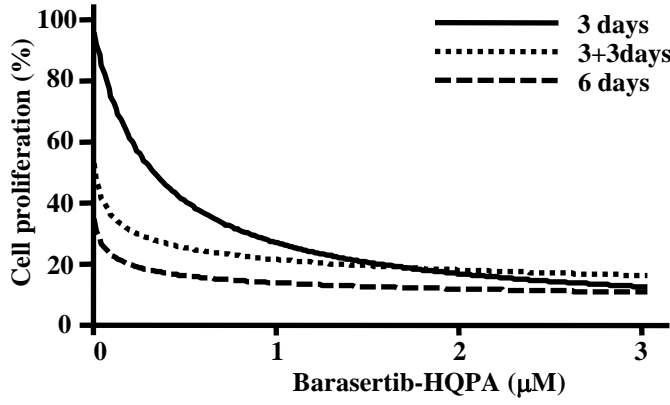

LND1

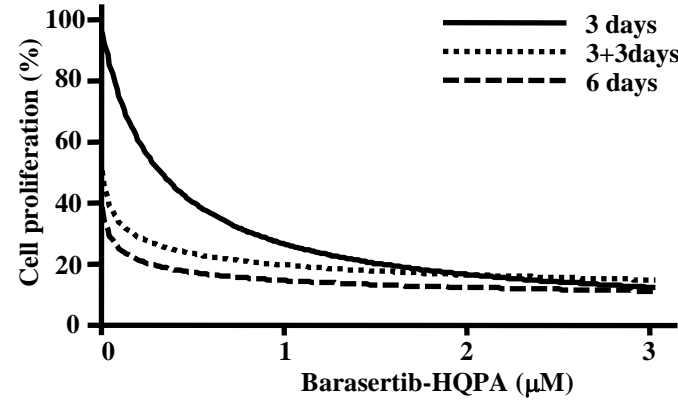

Supplement: Additional file 1: Figure S1. — Vemurafenib and barasertib-HQPA activity in melanoma cell panel. A. Melanoma cells were incubated with vemurafenib, ranging between 0.1 and 100 μM and the survival of cells was determined using MTT assay. The results are showed as dose/cell growth inhibition plots of the mean of three different experiments, evidencing that mutated cells are more sensitive to the drug. B. Melanoma cells were incubated with Barasertib-HQPA, ranging between 3 nM and 3 μM and for increasing time. The survival of cells was determined using direct cell count. The results are showed as drug cell proliferation inhibition/dose plots of the mean of three different experiments, evidencing that BRAF wt cells are more sensitive to the drug than the BRAF mutated ones. [file 12967_2015_385_MOESM1_ESM.pdf]

Fig. 2S

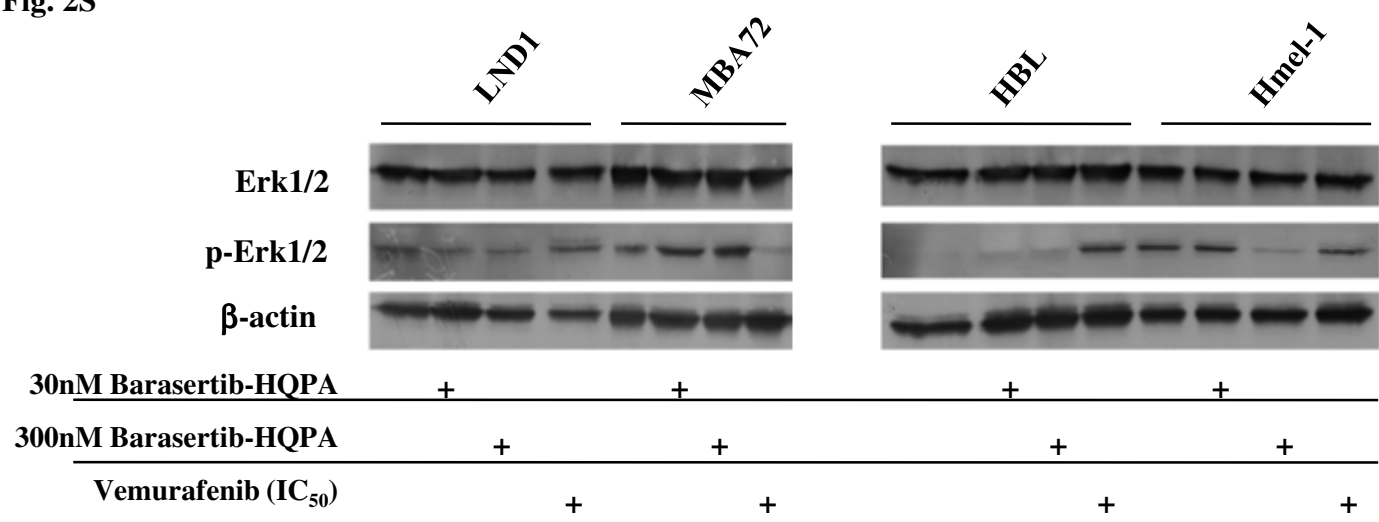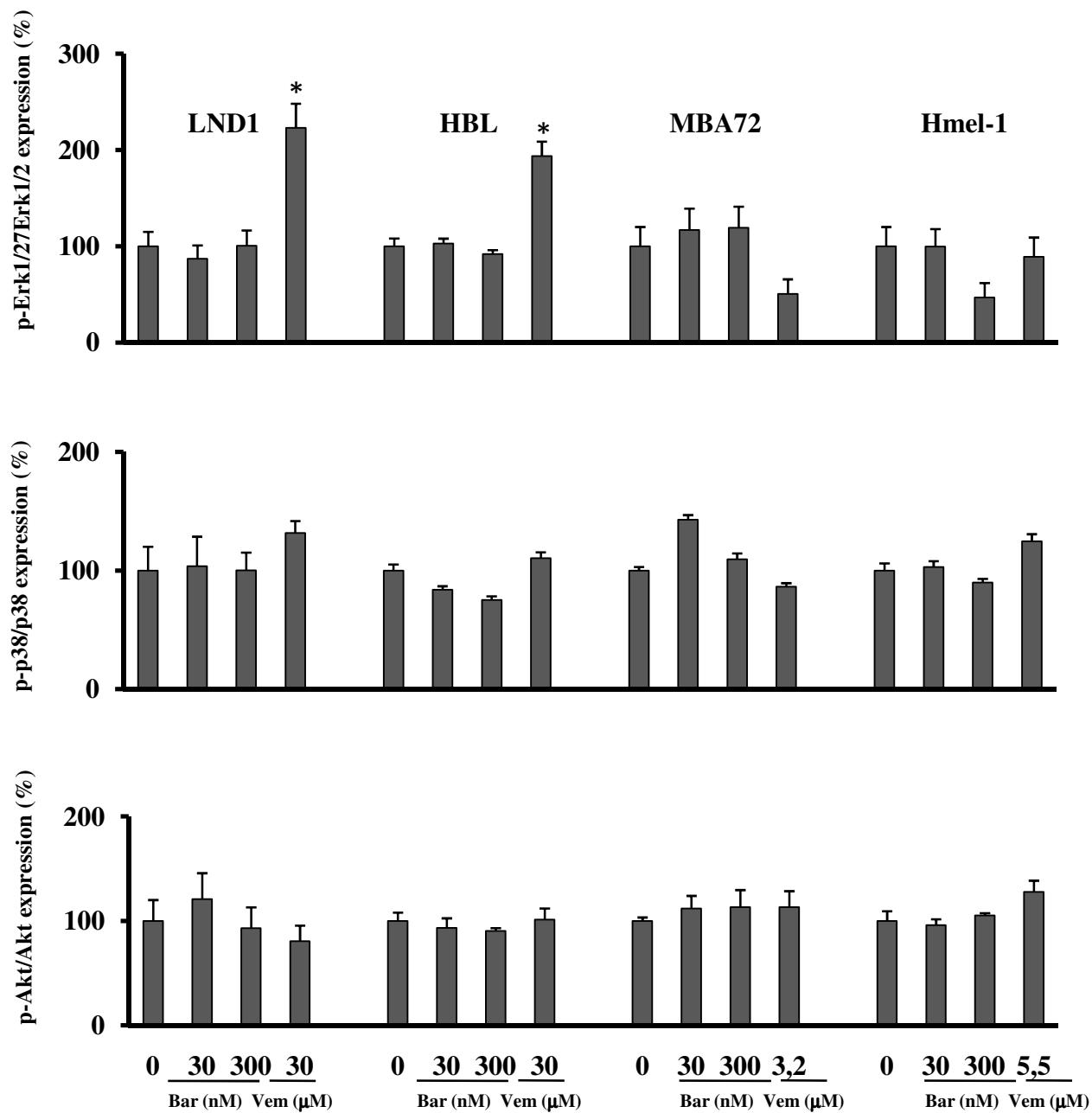

Supplement: Additional file 2: Figure S2. — Effect of barasertib-HQPA and vemurafenib on cell transduction pathways. Both BRAF-mutated and wt melanoma cells were incubated with barasertib-HQPA (Bar) and vemurafenib (Vem) for 3 days. The protein extracts from all samples were analysed by western blot utilising the β-actin to normalize the values and are quantified as phosphorylated form as respect the total one. A. Bands of p-Erk1/2 expression level from all samples are reported together with the quantification. Histograms are means of at least three different experiments. *p < 0.05 vs untreated cells. *p < 0.05 vs untreated cells. [file 12967_2015_385_MOESM2_ESM.pdf]

**Fig. 3S**

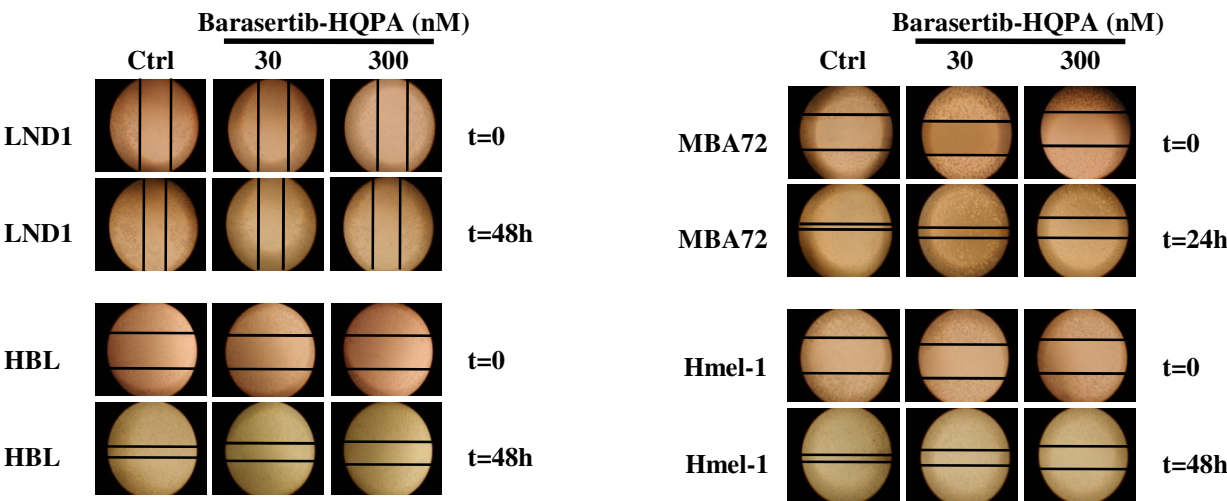

Supplement: Additional file 3: Figure S3. — Barasertib-HQPA ability to modulate cellular motility. Wound healing assay was performed at 24-48 h with 30 and 300 nM barasertib-HQPA. [file 12967_2015_385_MOESM3_ESM.pdf]

Fig. 4S

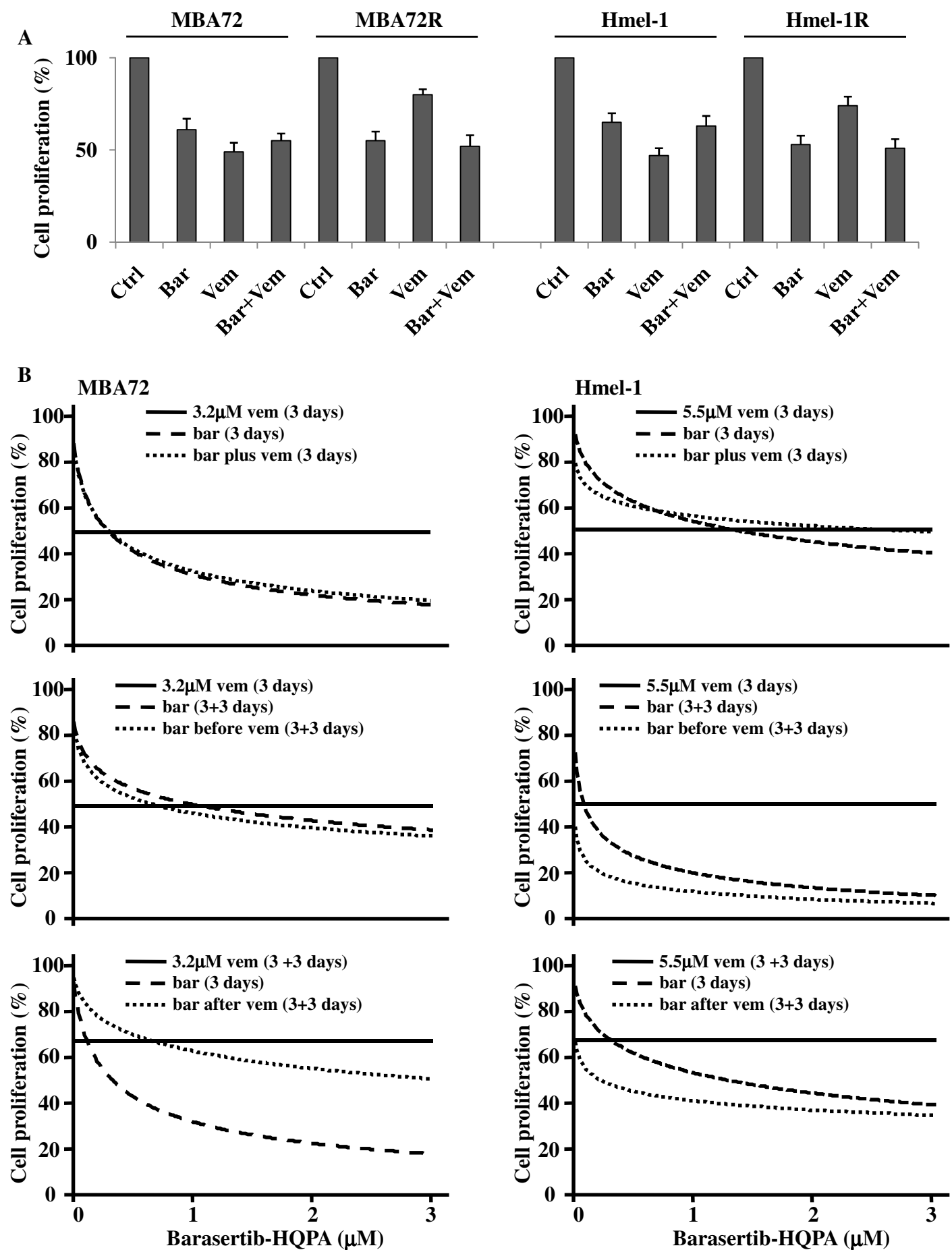

Supplement: Additional file 4: Figure S4. — Barasertib-HQPA plus vemurafenib in mutated melanoma cells. A. MBA72R and Hmel-1R were incubated with 300 nM barasertib-HQPA (Bar) and/or Vemurafenib (Vem: at 3.2 and 5.5 μM in MBA72R and Hmel-1R, respectively) for 3 days. The proliferating cells were determined using the direct cell count. Histograms are means of at least three different experiments. B. MBA72 and Hmel-1 cells were incubated with barasertib-HQPA plus vemurafenib given in three schedules, simultaneous (3 days) and 3 days-barasertib-HQPA (3, 30, 300, 3000 nM) before or after 3daysvemurafenib, and the survival of cells was determined using the direct cell count. [file 12967_2015_385_MOESM4_ESM.pdf]

**Fig. 5S**

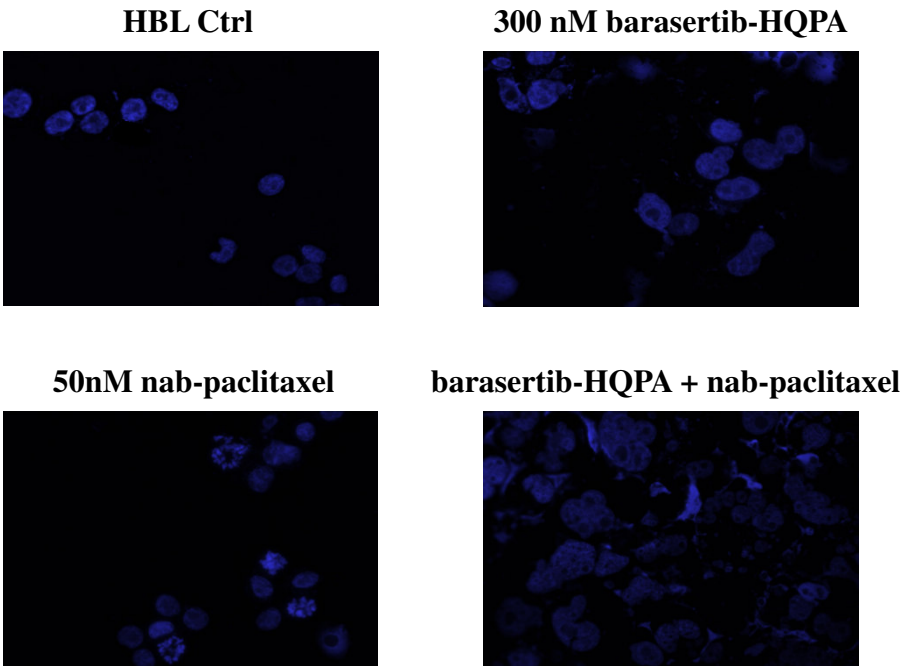

Supplement: Additional file 5: Figure S5. — Barasertib-HQPA plus nab-paclitaxel in melanoma cell panel. HBL cells exposed to 300 nM barasertib-HQPA and 50 nM nab-paclitaxel, alone or in combination, and polynucleate cells were evidenced by ICC (blue: DAPI). [file 12967_2015_385_MOESM5_ESM.pdf]
